# Iron Status in Neonates and Its Impact on Their Health Status at Birth

**DOI:** 10.7759/cureus.84434

**Published:** 2025-05-19

**Authors:** Osama M El-Asheer, Mohamed Gamil M Abo Elela, Heba G Ali, Ahmed M Hashem, Noha ElGyar

**Affiliations:** 1 Pediatric Medicine, Faculty of Medicine, Assiut University, Assiut, EGY; 2 Neonatology, Faculty of Medicine, Assiut University, Assiut, EGY; 3 Public Health, Faculty of Medicine, Assiut University, Assiut, EGY

**Keywords:** anemia, egypt, health status, iron status, neonates

## Abstract

Background: Iron is a vital micronutrient that performs a crucial role in numerous cellular processes. We aimed to evaluate the prevalence of anemia and the iron levels in newborns and their effect on overall health at birth, to link iron levels in newborns with maternal and newborn traits, and to identify predictors of ferritin levels in newborns.

Methods: This cross-sectional study comprised 100 mothers and their neonates, who were delivered either vaginally or via caesarean section, at Assiut University Hospital in Egypt. During the presentation for delivery, information about the mother's medical history was briefly documented, including her age, parity, gravidity, pregnancy-related complications, and blood samples were taken for a complete blood count (CBC). The neonates were assessed based on the following factors: gender, Apgar scores at first and fifth minutes, birth weight, need for admission to the neonatal intensive care unit (NICU), and neonatal cord blood samples were taken for CBC and serum ferritin analysis.

Results: At birth, three (3%) neonates were anemic, 15 (15%) neonates had low iron stores, and about three-fourths (74%) of mothers had iron deficiency (ID) anemia. Maternal anemia, neonatal low birth weight, and prematurity were predictors of ferritin level among the studied neonates.

Conclusions: ID is not excluded by normal haemoglobin levels. Pregnant women frequently suffer from anaemia. Maternal anemia is significantly associated with low serum ferritin in newborns. Therefore, to prevent unfavourable consequences for both the mother and the fetus, anemia during pregnancy should be appropriately diagnosed and treated. Given the significance of adequate iron reserves at birth for maintaining iron homeostasis during the early stages of infancy, all newborns should be routinely screened for haemoglobin levels and iron status at delivery. It is also highly recommended that serum ferritin be assessed in preterm neonates to determine their iron status.

## Introduction

Iron plays a vital part in various essential cellular processes, including oxygen transport, DNA synthesis, and muscle metabolism. Iron plays a crucial role in the early growth, development, and functioning of the human brain due to its significance in neuronal and glial energy metabolism, neurotransmitter production, and myelination [1].

Anemia is primarily caused by iron deficiency (ID), which is the most widespread nutritional disorder globally, impacting 33% of non-pregnant women, 40% of pregnant women, and 42% of children worldwide [2]. Pregnancy triggers a significant surge in iron requirements due to the enlarging maternal blood volume and the growing fetus. Pregnancy is characterized by a heightened risk or an already existing state of ID. Fortunately, when a mother has a mild ID, the body prioritizes the fetus's need for iron. During moderate and severe ID, the entire maternal-placental-fetal unit develops a severe ID with considerable short and long-term implications for the fetus [1].

ID during the fetal or early postnatal periods can impact brain chemistry, structure, and cognitive performance. Iron supplementation cannot cure the long-term motor and cognitive damage that can result from ID, which can occur with or without anemia [3].

One sensitive indicator to measure the body's iron status is serum ferritin, which is linked to iron reserves and is the primary protein in the liver that stores iron [4]. Previous studies assessing serum cord ferritin of neonates concluded that 16% and 17% of neonates had low ferritin levels [5,6].

Infants at risk for ID include those who were born prematurely, have a low birth weight, are small for their gestational age, or are the children of mothers with diabetes [6].

Previous research has found that maternal ID during pregnancy may be associated with an increased risk of adverse effects on newborns. A mother's ID results in a decrease in the baby's iron stores and increases the risk of ID in the first few months following childbirth [5]. Previous research on Egypt has shown that maternal anemia is a considerable risk factor for poor neonatal birth weight and blood-related health indicators [7,8].

This study aimed to assess the impact of maternal anemia on neonatal iron status, as measured by cord blood serum ferritin. A secondary objective was to identify additional maternal and neonatal factors associated with low neonatal ferritin levels.

## Materials and methods

Study setting and design

A cross-sectional hospital-based analytic study was performed at Assiut University Hospital in Upper Egypt. Information gathering took place between June 2023 and August 2023.

A total of 100 pregnant women and their newborns were enrolled at the time of delivery at the Woman's Health Hospital, part of Assiut University Hospitals, for the current research. The study included both mothers and their neonates who were born at Women's Health Hospital, whether the birth was a caesarean section or a normal vaginal delivery.

Sample size and technique

Version 7.2.01 of the Epi Info statistical program (Centers for Disease Control and Prevention (CDC), Atlanta, GA, USA) was employed to determine the sample size. The sample size was estimated using the following parameters: a 95% confidence level, a 5% margin of error, and the prevalence of anaemia in newborns (5.7%) [9]. The sample size was increased to 100 neonates from the initial 83 after a 10% nonresponse rate was included. The convenience (non-probability) sample was employed to find research participants.

Data collection 

Any newborn with a history of genetic or chromosomal disorder, a history of inborn errors of metabolic disease, a congenital deformity, or a neonate for whom we were unable to get a cord blood sample right after birth was excluded.

When the mother was presented for birth, a short history was collected, which included age, parity, gravidity, pregnancy-related complications (as gestational diabetes mellitus, preeclampsia, antepartum hemorrhage and premature rupture of membranes), and mode of delivery. For a complete blood count, prepartum mothers' blood samples were taken.

All neonates were evaluated as following: gender, Apgar score at first and fifth minutes, birth weight, gestational age and need for NICU admission. Samples of cord blood from newborns were collected for a full blood count and serum ferritin analysis.

Neonatal cord blood sample and biochemical analyses

Blood from the umbilical cord was collected at the time of delivery. The placenta was placed in a sterilized container with the umbilical cord securely fastened. The placenta was then handed over to the NICU staff for blood sampling. The umbilical vein, at its attachment to the placenta, was prepared with povidone iodine and left to dry for approximately one second.

A 10 ml sterile syringe with an 18-gauge needle was used for venous puncture. To avoid collapse of the vessel wall, the needle was inserted at an angle, with the bevel facing downwards, as blood was being drawn into the syringe. Approximately 6 to 7 milliliters of blood were drawn into the syringe, and the laboratory tubes were then filled. The complete blood pictures were done using the CELL-DYN Ruby System (Abbott Laboratories, Abbott Park, IL, USA), a multi-parameter automated hematology analyzer using multi-angle polarized scatter separation (MAPSS) optical technology for differentiating and analyzing blood cells. Lysing reagent CD 3200 CN FREE and scatter reagent CD 3200 were used.

The ADVIA Centaur XP system (Siemens Healthineers, Erlangen, Germany), an automated clinical chemistry analyzer, was used to measure cord ferritin. It uses direct chemiluminescence to detect analytes in the sample. It uses LH lite reagent and LH solid phase reagent.

Based on cord ferritin level, neonates were classified into neonates with normal ferritin level (25-200 ng/ml), neonates with low ferritin level (below 25 ng/ml) and neonates with high ferritin level (above 200 ng/ml) [5]. Maternal anemia was diagnosed if the mother’s haemoglobin (Hb) level was below 11 g/dl [10]. Anemia in neonates was defined as a Hb concentration < 13 g/dl [11].

Ethical approval

The study was approved by the Ethical Committee of the Faculty of Medicine at Assiut University with IRB and registered on clinicaltrials.gov with NCT05513105. The purpose of the study was explained to mothers before participation in the study. Informed consent was obtained from all participants before the interview, ensuring that their involvement would not affect their clinical services or therapy. Privacy and confidentiality of all data were guaranteed. The whole research was conducted according to the Helsinki Declaration.

Statistical analysis

SPSS v28 (IBM Corp., Armonk, NY, USA) was employed to conduct the statistical analysis. The normality of the data distribution was examined using the Shapiro-Wilks test. The independent sample t-test was utilized to compare means between groups, and quantitative parametric data were shown as mean and standard deviation (SD). The median and range of quantitative non-parametric values were displayed. The chi-square test was used to assess the qualitative variables, which were shown as frequency and percentage (%). When there were fewer than five cells, the Fisher exact test was employed. The correlation between continuous variables was tested using Spearman correlation. Multivariate linear regression analysis was used to identify the factors that predicted the ferritin level in newborns. P values < 0.05 are regarded as significant.

## Results

Table 1 shows that 15 neonates (15%) had low ferritin levels and 85 of them (85%) had normal or high ferritin levels. The mean age of mothers was 31.34 ± 4.65 years and the majority of mothers were from rural areas (89%). About three-fourths of them were multigravida and 60% of them delivered by vaginal delivery. Preterm rupture of membranes (PROM), antepartum postpartum hemorrhage (APH) and preclampsia occurred in about 24%, 21% and 10% of mothers. Maternal anemia was detected in 74% of mothers. There was a substantial association between low ferritin levels in neonates and the presence of PROM, APH and anemia in mothers. Neonates with lower ferritin levels had lower maternal Hb levels than neonates with normal or high ferritin levels and this was significant (p value=0.007).

**Table 1:** Association between neonatal ferritin level and maternal characteristics and maternal risk factors. Numbers, percentages (%), and mean ± SD were used to display the data. When the number of cells was fewer than five, the Fisher exact test was employed, and the chi-square test was used to examine the proportions across groups. The means of the groups were compared using the independent sample t-test. * P values < 0.05 are considered significant. Hb: haemoglobin, GDM: gestational diabetes mellitus, APH: antepartum haemorrhage, PROM: premature rupture of membranes.

Variables		Total	Level of ferritin		Statistical test	P value
			Low (n= 15)	Normal or High (n=85)		
Maternal characteristics						
Age (year)		31.34±4.65	30.20±5.1	31.54±4.57	-1.028	0.306
Residence	Rural	89 (89%)	14 (15.7%)	75 (84.3%)	0.338	0.999
	Urban	11 (11%)	1(9.1%)	10 (90.9%)		
Gravidity	Primigravida	29 (29%)	5 (17.2%)	24 (82.8%)	0.161	0.760
	Multigravida	71 (71%)	10 (14.1%)	61 (85.9%)		
Mode of delivery	Vaginal delivery	60 (60%)	8 (13.3%)	52 (86.7%)	0.327	0.568
	Cesarean section	40(40%)	7 (17.5%)	33 (82.5%)		
Maternal risk factors						
PROM	Yes	24(24%)	12 (50%)	12 (50%)	30.34	< 0.001*
	No	76(76%)	3 (3.9%)	73 (96.1%)		
APH	Yes	21(21%)	11 (52.4%)	10 (47.6%)	29.13	< 0.001*
	No	79(79%)	4 (5.1%)	75 (94.9%)		
Pre-eclampsia	Yes	10(10%)	1 (10%)	9 (90.0%)	0.218	0.999
	No	90(90%)	14(15.6%)	76 (84.4%)		
GDM	Yes	7(7%)	1 (14.3%)	6 (85.7%)	0.003	0.999
	No	93(93%)	14 (15.1%)	79 (84.9%)		
Mother’s anemia	Anemic	74 (74%)	15 (20.3%)	59 (79.7%)	6.20	0.01*
	Non anemic	26(26%)	0	26 (100%)		
Maternal Hb (g/dl)		10.46 ± 1.14	9.74±1.45	10.59±1.03	-2.75	0.007*

Table 2 reveals that about two-thirds of neonates were males, the majority of them were full-term and about three-fourths of them had normal birth weight. The mean neonatal Hb was 15.14 ± 1.18 g/dl while neonatal ferritin ranged from 10.01-309.4 ng/ml, with a median of 190 ng/ml. Neonatal ferritin levels were significantly associated with both birth weight and gestational age (p value=<0.001*). According to neonatal Hb level, three of the neonates (3%) were anemic and 97% of them were non-anemic. The mean of neonatal Hb level was lower in neonates with low ferritin level in comparison to those with normal or high ferritin and this was significant (p value<0.001). Other data show no significant difference between the different groups.

**Table 2:** Association between neonatal ferritin level and neonatal characteristics. When the number of cells was fewer than five, the Fisher exact test was employed, and the chi-square test was used to examine the proportions across groups. The means of the groups were compared using the independent sample t-test. * P values < 0.05 are considered significant. Hb: haemoglobin, NICU: neonatal intensive care unit

Variables		Total	Level of ferritin		Statistical test	P value
			Low (n= 15)	Normal or high (n=85)		
Neonatal gender	Male	65 (65%)	9 (13.8%)	56 (86.2%)	0.194	0.660
	Female	35 (35%)	6 (17.1%)	29(82.9%)		
Gestational age (weeks)	Mean±SD	38.09 ± 1.27				
	Pre-term(< 37 week)	20 (20%)	13 (65%)	7 (35%)	49.02	<0.001*
	Full term (37-40 week)	80 (80%)	2 (2.5%)	78 (97.5%)		
Birth weight (gm)	Mean±SD	2681.40 ± 307.599				
	Low birth weight (< 2500 gm)	23 (23%)	13 (56.5%)	10(43.5%)	40.39	<0.001*
	Normal birth weight (> 2500 gm)	77 (77%)	2 (2.6%)	75(97.4%)		
Apgar score	1^st^ minute	7.27 ± 1.87	6.93 ± 1.62	7.33±1.90	-0.757	0.541
	5^th^ minute	9.21 ± 0.76	9.33 ± 0.723	9.18± 0.779	-0.672	0.503
NICU admission	Yes	16 (16%)	3 (18.8%)	13(81.2%)	0.210	0.740
	No	84 (84%)	12 (14.3%)	72(85.7%)		
Neonatal Hb (g/dl)		15.14 ± 1.18	14.06 ± 1.04	15.33 ± 1.10	-4.14	< 0.001*
Neonates with anemia		3(3%)				
Neonates without anemia		97(97%)				
Neonatal ferritin (ng/ml) Median (range)		190 (10.01-309.4)				

Fetal cord ferritin had insignificant correlation with maternal age, parity, gravidity, maternal Hb, Apgar score, birth weight and gestational age as shown in Table 3.

**Table 3:** Correlation between cord ferritin, maternal and neonatal characteristics. Spearman correlation. r: correlation coefficient, p value significant if < 0.05.

Variables	r	p value
Maternal age	0.084	0.408
Parity	0.006	0.950
Gravidity	-0.082	0.418
Maternal hemoglobin	0.093	0.355
Apgar score-1^st^ minute	-0.078	0.443
Apgar score-5^th^ minute	-0.007	0.947
Birth weight	0.169	0.092
Gestational age	0.006	0.952

Table 4 displays that 63.3% of the variation in the ferritin level in neonates were explained by maternal Hb level, neonatal birth weight, gestational age, neonatal Hb level, APH and PROM (R square=0.633), but only maternal Hb, birth weight, gestational age and neonatal Hb level were the predictors of the ferritin level in neonates (p value=< 0.001, 0.02, 0.001 and 0.016 respectively).

**Table 4:** Predictors of ferritin level in the studied neonates. *: significant as P value < 0.05. Hb: haemoglobin, PROM: premature rupture of membranes, APH: antepartum hemorrhage, CI: confidence interval

Variables	Unadjusted linear regression			Adjusted linear regression (R square=0.633)		
	B	95%CI	P value	B	95%CI	P value
Maternal Hb g/dl	20.24	8.99: 31.49	0.001*	16.80	7.75:25.85	< 0.001*
Birth weight (reference=normal birth weight)	-98.48	-124.11: -72.85	0.001*	-40.88	-75.22: -6.54	0.02*
Gestational age (reference=full term)	-114.44	-139.58: -89.30	0.001*	-63.02	-98.04: -28	0.001*
Antepartum hemorrhage (reference =no APH)	-61.01	-92.12: -29.91	0.001*	-9.00	-33.80: 15.79	0.473
PROM (reference=no PROM)	-73.14	-101.44: -44.83	0.001*	-16.15	-41.30: 8.99	0.205
Neonatal Hb g/dl	28.67	18.65:38.69	< 0.001*	10.23	1.94:18.52	0.016*
Constant				0.034		

## Discussion

Iron is essential for the development of the placenta and fetus, as well as for the expansion of erythropoiesis. Anemia during pregnancy can result in severe complications for both the mother and the baby, including long-term complications such as low birth weight and low iron stores in infancy, which may contribute to childhood anemia and compromised development [12]. In order to identify determinants of neonatal ferritin levels, the current study evaluated the prevalence of anemia and the iron status of newborns at Assiut University Hospital in upper Egypt.

The current study found that the mean Hb of neonates was 15.14 ± 1.18 g/dl. This is in line with the findings of Tiruneh et al. [13], who demonstrated that the median cord haemoglobin value is 15 g/dL, with a range of 4.2 to 20 g/dL. The inclusion criteria for both preterm and term newborns, as well as underweight and normal-weight neonates who took part in these trials, were comparable, which may account for this commonality.

The prevalence of anemia in newborns was found to be 3% in the current research, which was less than the 9% found in an Ethiopian study [14]. Also, higher prevalence of newborn anemia had been reported from a previous study in Nigeria (28.9%) [15]. The discrepancies in the clinical characteristics of the study participants and the varied socioeconomic conditions of the participants could be the reasons for this difference. Another potential explanation for this discrepancy is that the sample size may have varied.

The present study found that about 15% of neonates have low ferritin levels. The difference in percentages between neonates with low ferritin levels (15%) and low Hb levels (3%) could be explained by the fact that there are three classes of iron deficiency based on severity: biochemical iron deficiency, iron-limited erythropoiesis, and biochemical iron deficiency with anemia. A low serum ferritin and iron level indicates biochemical iron deficiency, while a decrease in reticulocyte Hb content and mean corpuscular volume, but no change in Hb or hematocrit, are symptoms of iron-limited erythropoiesis [16]. This clarification highlights the significance of measuring ferritin level as a screening measure for the early stage of ID as ID at perilous times throughout growth of the brain is accompanied by long-standing neurocognitive complications.

Consistent with the findings of Mahdi et al. [5], who concluded that 16% of newborns had low ferritin levels, the current study found that 15% of neonates had low ferritin levels. Similarly, a previous Chinese study revealed that a total of 19.8% of newborns were iron deficient [17].

According to the results of the current study, 74% of mothers had anemia. This finding was greater than that found by a prior study carried out in Nigeria (40%); the reduced prevalence of anaemia in that study may be explained by the fact that only women with moderate to severe anaemia were recruited [18]. The fact that the prevalence of anemia in pregnancy varies among women with various socioeconomic backgrounds, lifestyles, health-seeking behaviours, iron supplementation during pregnancy, dietary practices during pregnancy, spacing between children, parity, and gravidity may also account for the variation in prevalence.

Regarding risk factors for low neonatal iron stores, the current study revealed significant association between low ferritin level and PROM (p value=<0.001). These results corroborated those of a previous study that concluded that PROM itself might affect iron status, potentially impacting the level of ferritin [19]. PROM can lead to preterm birth, potentially leading to reduced iron stores in newborns through reduced iron accumulation in the last trimester [20].

In the present study, it was found that there was a statistically significant association between low ferritin level, gestational age and birth weight (p value=<0.001).

This is consistent with another study that explored the factors affecting iron status in neonates which underlined that prematurity is a major risk factor for ID [20]. This study emphasized the combined effect of prematurity and low birth weight on ID, where prematurity interrupts iron transfer, and low birth weight further indicates a smaller maternal iron endowment, resulting in a higher risk of low neonatal ferritin levels.

The current study concluded that maternal anemia can be associated with ID in their newborns (p value=<0.01). In a similar vein, earlier studies that examined the prevalence of anemia and the relationships between maternal iron status, hepcidin, and neonatal iron status in neonates born to adolescents who were pregnant came to the conclusion that ID in their offspring could be linked to maternal anaemia and ID [11].

The current study revealed that cord ferritin levels in boys did not differ significantly from those in girls (p value=0.660). Prior Chinese research that examined the gender-specific relationship between serum ferritin and neurodevelopment in children aged six to 12 months found that the mean serum ferritin levels were considerably higher in female infants than in male infants. This finding contradicts that study. Nevertheless, the processes behind sex-related variations in ferritin levels throughout early childhood have not received much attention up to this point. Hormone-mediated variations in metabolism might account for this process. It is commonly known that male and female babies have differing levels of leptin and serum insulin [21].

Regarding the impact of low iron stores at birth, the current study revealed that there was no significant association between cord ferritin level and Apgar score at first minute and at fifth minute (p value=0.541 and 0.503, respectively). Zuliyana et al. concluded similar results and revealed that the initial health assessment (Apgar) may not be directly affected by decreased ferritin alone [22].

The current study revealed that fetal cord ferritin had an insignificant correlation with maternal age, parity, and gravidity. Similarly, Adediran et al. [23] support the absence of a direct correlation between ferritin and maternal age or parity, aligning with our results. However, Christensen et al. [24] discovered that serum ferritin was impacted by gestational age, with serum ferritin and iron marginally rising as gestational age rose.

Regarding the predictors of ferritin level among the studied neonates, the current study concluded that maternal Hb, neonatal birth weight, gestational age and neonatal Hb. In a similar vein, Teixeira et al. [25] found that maternal Hb level was an independent predictor of infants’ Hb levels. This is explained by the fact that the foetus receives its iron from the mother's transferrin, and when the mother's iron reserves are reduced, the fetus's ability to acquire iron is also reduced [14].

Regarding the gestational age as one of the predictors of ferritin level in the studied neonates (p value=0.016), similar findings were obtained in a prior study that indicated low blood ferritin levels were six times as common in preterm neonates as in term babies [26].

Strengths and limitations

This study has several strengths, including its focus on a clinically relevant topic in a resource-limited setting, the use of objective biochemical markers to assess both maternal and neonatal iron status, and the inclusion of term, appropriate-for-gestational-age neonates, which minimizes confounding due to prematurity or intrauterine growth restriction. Cord blood sampling offers a practical and minimally invasive approach that can be replicated in similar contexts. However, the study also has limitations. The cross-sectional design limits causal inference, and the single-center setting with a relatively small sample size may affect the generalizability of our findings. Importantly, we did not assess inflammatory markers such as C-reactive protein, which could influence ferritin levels and potentially confound interpretations of iron deficiency. We also highlight the importance of including maternal nutrition status, maternal dietary intake, socioeconomic level, maternal iron supplementation and maternal inflammation or infection in future research to more comprehensively assess their impact on ferritin levels. Future studies with larger, more diverse populations and a longitudinal design are recommended to validate and expand upon these findings.

## Conclusions

Anemia is a common health problem among pregnant women and it is a significant risk factor for low iron stores in neonates. Neonates with normal ferritin levels had significantly higher Hb levels compared to those with low ferritin, suggesting that better iron stores translate to better blood parameters. There is a significant association between low ferritin levels in neonates and occurrence of prematurity and low birth weight. Normal Hb levels don’t exclude ID among neonates.

We recommend further longitudinal studies to test the feasibility and the benefit of routine neonatal ferritin testing for early detection of neonatal anemia. Maternal anemia must be adequately prevented and managed through comprehensive antenatal care and the integration of hematological assessments into routine prenatal checkups. In prenatal care follow-up, it is preferable to appropriately inform and counsel women about the symptoms of anemia and the significance of iron supplementation throughout pregnancy. Future multicenter studies on large number of neonates with longer duration of follow-up are highly warranted.
